# Incidence, characteristics and risk factors of adverse drug reactions in hospitalized children – a prospective observational cohort study of 6,601 admissions

**DOI:** 10.1186/1741-7015-11-237

**Published:** 2013-11-07

**Authors:** Signe Thiesen, Elizabeth J Conroy, Jennifer R Bellis, Louise E Bracken, Helena L Mannix, Kim A Bird, Jennifer C Duncan, Lynne Cresswell, Jamie J Kirkham, Matthew Peak, Paula R Williamson, Anthony J Nunn, Mark A Turner, Munir Pirmohamed, Rosalind L Smyth

**Affiliations:** 1 Department of Women’s and Children’s Health, Institute of Translational Medicine (Child Health), University of Liverpool, Alder Hey Children's NHS Foundation Trust, Eaton Road, Liverpool L12 2AP, UK; 2 Department of Biostatistics, University of Liverpool, Shelley’s Cottage, Brownlow Street, Liverpool L69 3GS, UK; 3 Research and Development, Alder Hey Children’s NHS Foundation Trust, Eaton Road, Liverpool L12 2AP, UK; 4 Oncology Unit, Alder Hey Children’s NHS Foundation Trust, Eaton Road, Liverpool L12 2AP, UK; 5 MRC Biostatistics Unit, Institute of Public Health, University Forvie Site, Robinson Way, Cambridge CB2 0SR, UK; 6 Department of Women’s and Children’s Health, University of Liverpool, First Floor, Liverpool Women’s Hospital, Crown Street, Liverpool L8 7SS, Uk; 7 The Wolfson Centre for Personalised Medicine, Department of Molecular and Clinical Pharmacology, Institute of Translational Medicine, University of Liverpool, Block A: Waterhouse Building, 1-5 Brownlow Street, Liverpool L69 3G, UK; 8 Institute of Child Health, University College London, 30 Guilford Street, London WC1N 1EH, UK

**Keywords:** Adverse drug reactions, Drug safety, Hospitalized children, Risk factors, General anesthesia, Peri- and post-operative pain management

## Abstract

**Background:**

Adverse drug reactions (ADRs) are an important cause of harm in children. Current data are incomplete due to methodological differences between studies: only half of all studies provide drug data, incidence rates vary (0.6% to 16.8%) and very few studies provide data on causality, severity and risk factors of pediatric ADRs. We aimed to determine the incidence of ADRs in hospitalized children, to characterize these ADRs in terms of type, drug etiology, causality and severity and to identify risk factors.

**Methods:**

We undertook a year-long, prospective observational cohort study of admissions to a single UK pediatric medical and surgical secondary and tertiary referral center (Alder Hey, Liverpool, UK). Children between 0 and 16 years 11 months old and admitted for more than 48 hours were included. Observed outcomes were occurrence of ADR and time to first ADR for the risk factor analysis.

**Results:**

A total of 5,118 children (6,601 admissions) were included, 17.7% of whom experienced at least one ADR. Opiate analgesics and drugs used in general anesthesia (GA) accounted for more than 50% of all drugs implicated in ADRs. Of these ADRs, 0.9% caused permanent harm or required admission to a higher level of care. Children who underwent GA were at more than six times the risk of developing an ADR than children without a GA (hazard ratio (HR) 6.40; 95% confidence interval (CI) 5.30 to 7.70). Other factors increasing the risk of an ADR were increasing age (HR 1.06 for each year; 95% CI 1.04 to 1.07), increasing number of drugs (HR 1.25 for each additional drug; 95% CI 1.22 to 1.28) and oncological treatment (HR 1.90; 95% CI 1.40 to 2.60).

**Conclusions:**

ADRs are common in hospitalized children and children who had undergone a GA had more than six times the risk of developing an ADR. GA agents and opiate analgesics are a significant cause of ADRs and have been underrepresented in previous studies. This is a concern in view of the increasing number of pediatric short-stay surgeries.

## Background

Adverse drug reactions (ADRs) are an important cause of iatrogenic morbidity and mortality in patients of all ages [1-5]. ADRs in children may differ from those in adults due to age-dependent physiological characteristics which affect the pharmacokinetics and pharmacodynamics of drugs [1,3,6,7].

A recent systematic review of 102 studies of ADRs in children by Smyth *et al*. [8] showed that previous studies have differed widely in their definition of ADRs, clinical settings, and age range of children studied and a high proportion had major shortcomings in design and/or reporting. A large proportion did not report data on incidence, severity and causality of ADRs or drugs and reaction types implicated. Study sizes for the 21 prospective pediatric inpatient studies ranged from 81 to 3,726 patients; three of these 21 studies were large (n >1,000). Reported incidence rates for hospitalized children experiencing an ADR ranged from 0.6% to 16.8%. A recent prospective analysis of 3,695 patient-episodes in adults [5] reported an ADR incidence rate of 14.7% with estimated rates in earlier studies ranging from 0.86% [9] to 37% [10] depending on study population, design and setting.

Data on the drugs associated with ADRs were only available in 52 of 102 studies investigated in the systematic review by Smyth *et al*. and many did not report the associated clinical presentations. Although 70% of the studies analyzed in this systematic review referred to a causality assessment, less than one third reported this in detail. Of 34 studies which assessed the severity of ADRs, only 20 provided a reference for the assessment tool used, with proportions of severe reactions reported from 0 to 66.7%. Only 14 studies provided data on the avoidability of ADRs and outcomes differed widely, with 7% to 98% of ADRs deemed definitely/possibly avoidable. Furthermore, few studies to date have investigated risk factors for ADRs in children. In the systematic review, female gender (10/19 studies), increasing number of drugs (16/17 studies), off-label use (3/3 studies) and oncological treatment (2 studies) were identifiable risk factors [8]. More recently, Rashed *et al*. conducted a prospective multicenter study of 1,278 hospitalized children (1,340 admissions) and, using multivariate logistic regression, found that multiple drug use and older age were risk factors for ADRs [2].

Reducing the impact of pediatric ADRs requires precise estimates of the incidence and nature of ADRs. Given the discordances in the extant literature, we designed a study large enough and of sufficiently robust design to overcome the problems identified in the previous literature. The aim of this study was to determine the incidence of ADRs in pediatric medical and surgical inpatients, to characterize those ADRs identified in terms of type, medication implicated, causality, and severity, and to identify factors which increase the risk of ADRs.

## Methods

### Study design and setting

The study was conducted over one year in a single secondary and tertiary pediatric referral center, Alder Hey Children’s National Health Service (NHS) Foundation Trust, which treats 200,000 children a year from the North West of England, North Wales, Shropshire and the Isle of Man. The Accident and Emergency department treats more than 60,000 children every year. There are 274 inpatient beds, including a pediatric ICU (PICU). Although neonates were included in the study, the hospital does not have a designated neonatal intensive care unit (NICU) (since a NICU exists in a nearby tertiary maternity center). Any neonates requiring surgical management (including cardiac surgery) are transferred to Alder Hey and cared for on the PICU (if ventilated), the cardiac ward, or on the neonatal ward (surgical patients requiring ventilation). The study population comprised children between 0 and 16 years 11 months old on admission, who were inpatients between 1 October 2009 and 30 September 2010. Extensive pilot work before the study established that the study team did not have the resources to carry out a detailed review of every inpatient every day. In 2008, a total of 39,747 inpatient admissions were recorded (emergency admissions, elective admissions and day-case attendances); of those 10,943 stayed longer than 24 hours and 5,357 stayed longer than 48 hours. A pragmatic decision was thus made to include only those children who had been inpatients for >48 hours.

Admissions included in this study were elective and emergency admissions of all pediatric medical and pediatric surgical specialities. Observations were carried out on 17 wards, including oncology wards and the high dependency unit (HDU). Patients were not observed while admitted to the PICU, theater, recovery or the department of radiology. We used the established hospital database for the recruitment of patients to the study. Electronic files containing a list of all children in the study population who met the inclusion criteria were automatically generated every 12 hours. Each child was followed up every 48 or 72 hours on weekdays and weekends, respectively, by one member of a multidisciplinary team of researchers comprising two research pharmacists, one research nurse and a pediatrician. We recorded details of all drugs administered on the wards, occurrence of new symptoms or those that had worsened and abnormal results that may have indicated the occurrence of an ADR, taking into account the case history, the ADR profiles of medication and the temporal relationship between drug exposure and reaction. Abnormal results included laboratory data and imaging reports, which are recorded electronically on the hospital database. These were routinely screened every time the patient was reviewed and any abnormal results recorded. We aimed to include all potential reactions to any medication administered in hospital (including those started prior to admission) and presenting after admission to a ward; each reaction was followed up with a detailed assessment by one research team member. Suspected reactions to certain blood products, total parental nutrition and intravenous hydration fluids were excluded from this study (Table 1).

**Table 1:** Excluded medicinal products

	**Excluded**	**Included**
Topical anesthetics	Lidocaine 2.5%, prilocaine 2.5% cream (EMLA^®^) or tetracaine 4% gel (Ametop^®^)	LAT gel (lidocaine 4% and adrenaline 0.1% and tetracaine 0.5% gel)
Ranitidine	Ranitidine added to TPN	Ranitidine administered otherwise
Heparin	Heparin administered as intermittent intravenous heparin flush.	Intermittent intravenous injection other than heparin flush, heparin administered as continuous intravenous infusion or as subcutaneous injection.
Total parenteral nutrition (TPN)	Total parenteral nutrition (TPN)	
Intravenous hydration fluids	Intravenous hydration fluids	Any drugs added to intravenous fluids
Rectal washouts	Rectal washouts with sodium chloride 0.9%	
Blood products	Red cells	Antithrombin III concentrate
	Platelets	Dried prothrombin complex
	Cryoprecipitate	Drotrecogin alfa (activated)
	Albumin solutions	Factor VIIa (recombinant)
	Fresh frozen plasma	Factor VIII fraction, dried
		Factor VIII inhibitor by-passing fraction
		Factor IX fraction, dried
		Factor XIII fraction, dried
		Protein C concentrate
Oxygen therapy	Oxygen therapy	

### ADR definition

We defined ADRs according to the definition of Edwards and Aronson: an ADR is ‘an appreciably harmful or unpleasant reaction, resulting from an intervention related to the use of a medicinal product, which predicts hazard from future administration and warrants prevention or specific treatment, or alteration of the dose regimen, or withdrawal of the product’ [11]. Prescribing errors, administration errors and intentional drug overdoses were thus not considered ADRs in this study.

### Causality and severity assessment of ADRs

The ADR case report was assessed independently by a research nurse, a research pharmacist and a pediatrician using the Liverpool ADR causality assessment tool as unlikely, possible, probable or definite [12]. Outcome reporting was based on consensus agreement among the three assessors; if agreement could not be achieved the case was referred to a panel of two of the senior investigators (MAT, RLS, AJN and MPir); each panel reached consensus about causality. Our estimate for the overall incidence was based on the sum of probable and definite ADRs only, as these ADRs are deemed to have a low probability of the underlying disease being responsible for the reaction. The severity of ADRs was assessed using the Hartwig scale [13]. Reactions classified as level four and above were considered severe.

### Incidence

Incidence was calculated in two ways by dividing: (i) the number of admissions in which at least one ADR occurred by the total number of admissions regardless of drug exposure; and (ii) the number of children with at least one ADR by the total number of children regardless of drug exposure.

### Risk factor analysis

Time from admission to first ADR was calculated in days. For patients admitted to PICU this was time to first ADR prior to PICU admission. If no ADR occurred prior to PICU admission, time from admission to first ADR was censored at the time of admission to PICU. ADRs occurring after PICU were included in the overall incidence calculation. For the analysis of risk factors, data collected for each patient during their first admission only were included. We assessed age, gender, number of drugs, receipt of a general anesthesia (GA) and oncology status as risk factors. Oncology patients were defined as those requiring on-going medical treatment for a malignancy of a solid organ or of the hematopoietic system. The number of drugs refers to the daily number of drugs administered to the patient on the ward. This risk factor was treated as a continuous, time-varying covariate in the multivariate model. The factor ‘received a GA’ was considered to be present from the first day the patient received a GA until discharge from the hospital. This risk factor was treated as a binary, time-varying variable in the multivariate model that takes the value zero on days up to the GA and one thereafter for the remaining days of a patient’s admission.

### Statistical methods

Time to first ADR was compared between groups using a log-rank test (extending to a log rank test for trend when appropriate) and Kaplan-Meier curves estimated. A Cox proportional hazards regression model for an ADR was fit to the data. Results are given in terms of the hazard ratio (HR) and associated 95% confidence interval (95% CI). Due to their clinical importance, all of the risk factor variables are included in a multivariate model. The assumptions of the model were assessed as follows. The proportional hazards assumption for each covariate was investigated using log cumulative hazard plots and Schoenfeld residual plots. The assumption was also tested by including a time-dependent covariate effect. Deviance residuals were plotted against the linear predictor to look for mis-modelling of the data and empirical validation of the model was done using a data splitting technique to assess model accuracy. Patients with missing prescription details for the entire duration of the admission were excluded from the analysis. The inclusion of patients with partially missing prescription details (for example, prescription details for day of discharge) was assessed on a case-by-case basis. They were only included if it was considered unlikely that this would have led to missing an ADR. Furthermore, any potentially missed ADRs towards the end of the stay are unlikely to have had an impact on the risk factor analysis as we used time to first ADR as the observed outcome. Investigations were based on clinical indication. For example, hypertension could only be identified if a child’s blood pressure was monitored for clinical reasons. We recorded ADRs observed between 1 October 2009 and 30 September 2010. Patients admitted between 28 and 30 September 2009 or discharged after 30 September 2010 who experienced an ADR before 1 October 2009 or after 30 September 2010, respectively, were counted as admissions without ADR in the analysis. Consequently, there are 180 admissions that lie outside the observation period where an ADR may have occurred that has not been recorded. All statistical analysis was carried out using the statistical software package R (version 2.13.2) using a two-sided significance level of 0.05 (5%) throughout.

### Reporting

This study was reported according to the Strengthening the Reporting of Observational Studies in Epidemiology (STROBE) guidelines [14].

### Ethics

This study used routinely collected clinical data in an anonymized format. The Chair of the Liverpool Paediatric LREC informed us that this study did not require individual patient consent or review by an Ethics Committee.

## Results and discussion

### Participants and descriptive data

**Figure 1:**
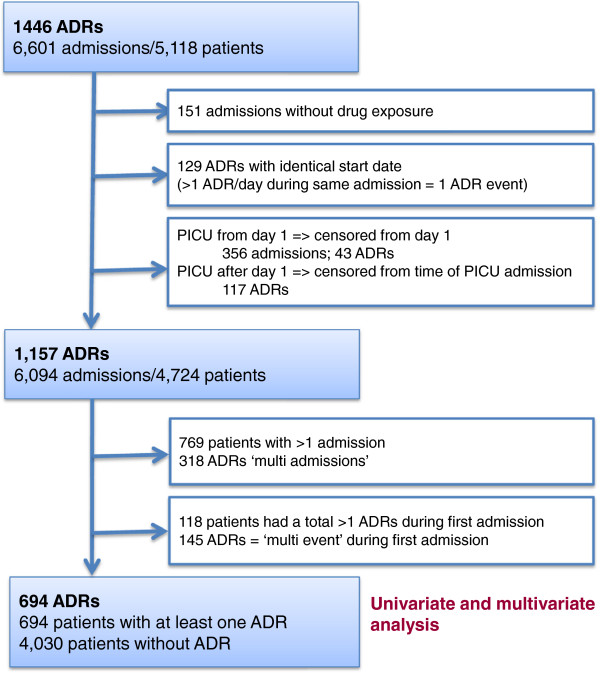
Flowchart outlining the number of admissions included in the univariate and multivariate risk factor analysis.

A total of 6,825 eligible admissions were identified. Of these, 181 (2.7%) admissions could not be included due to missing data. Forty-three patients spent their entire admission on PICU and were thus excluded. Consequently, 6,601 admissions of 5,118 children were included in the study; of these, 827 were also admitted to PICU with 45.2% being cardiology or cardiothoracic patients. The median length of follow up time across admissions was five days (interquartile range (IQR) 3 to 8 days, range 2 to 280 days). The median age on admission was 3.4 years (IQR 0.6 to 10.7); 2,297 (44.8%) were female. A total of 4,284 (83.7%) children had one admission and 834 children had more than one admission. A total of 2,856 children (55.8%) underwent at least one GA during 3,265 admissions (49.4%); 114 children (2.2%) were oncology patients. In total, 2,934 suspected ADRs were assessed. After causality assessment, 213 (7.3%) of the suspected ADRs were deemed definite, 1,233 (42.0%) probable, 896 (30.5%) possible and 592 (20.2%) unlikely. Consensus was reached independently in 1,805 cases (61.5%) and by panel decision in 1,128 cases (38.5%). All definite and probable ADRs were included in the further analysis (total number 1,446; Figure 1). The overall incidence of definite and probable ADRs based on admissions was 15.9% (95% CI 15.0 to 16.8) and 17.7% when based on numbers of patients (95% CI 16.7 to 18.8). The ADR incidence for patients with only one admission was 14.7% (95% CI 13.7 to 15.9). For patients with more than one admission, the incidence per admission was 18.0% (95% CI 16.4 to 19.6) but 32.7% per patient (95% CI 29.6 to 35.9). A total of 0.9% of the ADRs were severe and required patient transfer to a higher level of care. One patient sustained permanent harm (peripheral neuropathy due to vincristine). No ADR resulted in death. Details of all severe reactions by reaction type and associated drugs are listed (Tables 2 and 3). Common ADR types and ADRs observed following GA are summarized in Table 4.

**Table 2:** Assessment of severity using the Hartwig severity scale

**Severity level**	**Description**	**Number of ADRs at each severity level**^**a**^	
		**Number**	**%**
1	Required no change in treatment	322	22.3%
2	Drug dosing or frequency changed	66	4.6%
3	Required treatment or drug administration discontinued	1,046	72.3%
4	Resulted in patient transfer to higher level of care	12	0.8%
5	Caused permanent harm to patient or significant hemodynamic instability	1	0.1%
6	Directly or indirectly resulted in patient death	0	0%

^a^Denominator was the total number of probable or definite ADRs. ADRs, adverse drug reactions.

**Table 3:** Severe reactions (Hartwig scale ≥4) by reaction type and medication implicated

**Severity level**	**ADR type (count)**	**Medication implicated (count)**	**Admission to PICU/ HDU (if > once)**
4	Cardiac failure (1)	Bisoprolol (1), Carvedilol (1)^a^	HDU
	Sedation withdrawal (1)^b^	Fentanyl (1), Midazolam (1), Promethazine (1) , Chloral hydrate (1)	PICU
	Raised INR and hemorrhage (1)	Warfarin (1)	HDU
	Pulmonary edema (1)	Diazoxide (1)	HDU
	Respiratory depression (5)	Fentanyl (4), Ketamine (2), Midazolam (1),	PICU (3)^c^ HDU (2)
	Respiratory arrest (2)	Fentanyl (2), Sevoflurane (1), Isoflurane (1), Ketamine (1)	PICU, HDU
5	Peripheral neuropathy (1)	Vincristine (1)	N/A

^a^Both drugs caused significant fluid retention in this patient; ^b^this patient had been on sedating drugs for 10 days by the time withdrawal became clinically significant; ^c^ADR was not the only factor leading to PICU admission; other clinical factors may also have contributed. *ADR* adverse drug reaction, *HDU* high dependency unit, *INR* international normalized ratio, *PICU* pediatric ICU.

**Table 4:** Common ADR types observed

**Reaction type**	**All reactions**		**Reaction following GA**^**b**^	
	**N**	**% of all reactions**^**a**^	**N**	**% of reaction types, where reaction followed GA**
Nausea and/or vomiting	400	27.5%	295	73.8%
Pruritus	243	16.7%	232	95.5%
Constipation	155	10.6%	107	69.0%
Diarrhea (9/88 with vomiting)	88	6.0%	0	0.0%
Somnolence (without cardio-respiratory symptoms)	50	3.4%	34	68.0%
Respiratory depression (41)/arrest (3)	44	3.0%	43	97.7%
Candidiasis	41	2.8%	0	0.0%
Urinary retention	40	2.7%	37	92.5%
Rash	31	2.1%	3	9.7%
Hypokalemia	25	1.7%	0	0.0%
Hypotension	22	1.5%	9	40.9%
Hepatotoxicity(6)/increased transaminases (12)^c^	18	1.2%	1	5.6%
Stomatitis	16	1.1%	0	0.0%
Myoclonus	15	1.0%	14	93.3%
Pancytopenia	13	<1%	0	0.0%
Hyperglycemia	12	<1%	0	0.0%
Hypertension	11	<1%	2	18.2%
Allergic reactions	10	<1%	3	30.0%
Pain (4/10 pain in jaw, 2/10 back pain)	10	<1%	0	0.0%
Other reactions ( occurred <10 times)	213	14.6%	65	30.5%
Total	1,457		845	58.0%

^a^If the same patient experienced two types of reactions to the same medication(s) at the same time this would have been reported as one ADR case but will be listed here as two reaction types, for example, a patient with respiratory depression and bradycardia = one ADR case, but is listed as two reactions; ^b^reaction occurred post theater AND drugs given in theater and/or used in post-operative pain management were implicated; ^c^transaminases were raised in all cases. Additionally, if other parameters of liver function such as bilirubin and INR were also raised, we classified this as hepatotoxicity. *ADR*, adverse drug reaction, *GA* general anesthesia; *N* number, *INR* international normalized ratio.

To our knowledge, this is the largest pediatric in-hospital study investigating ADRs. The study population represents a wide range of pediatric medical and surgical specialities, given the nature of the hospital as a regional center. Our methodology included causality and severity assessments using validated tools. An avoidability assessment was not undertaken because of the lack of appropriate tools and imperfect definitions of preventability as highlighted recently by Ferner and Aronson [15]. The most frequently used assessment tools were Schumock and Thornton [16] and Hallas [17], which are based on appropriateness of prescribing or treatment choice. These tools might be used successfully to improve prescribing practice in specific clinical circumstances. However, they become problematic when treatment is guided by multiple sources of tertiary pediatric specialist advice. Rashed *et al.*[2] reported an overall ADR incidence of 16.5 (95% CI 14.5 to 18.7) per patient in their multicenter study of 1,278 patients (1,340 admissions) and Davies *et al.*[5], who conducted a comparable study in adults, observed an incidence of 14.7% per episode (admission) and 15.8% per patient. However, both Rashed *et al.* and Davies *et al*. used the Naranjo algorithm [18] for causality assessment and included possible, probable and definite ADRs in their calculations. In our study, we only included probable and definite ADRs as these have a low probability of the underlying disease causing the reaction. Had we included possible ADRs, our overall ADR incidence rate would have been more than 25% per child (data not shown) which is much higher than in adults. One possible explanation is that many common medicines have not been tested properly or at all in infants and children.

### Reaction types, drug classes implicated in ADRs and risk factor analysis

**Figure 2:**
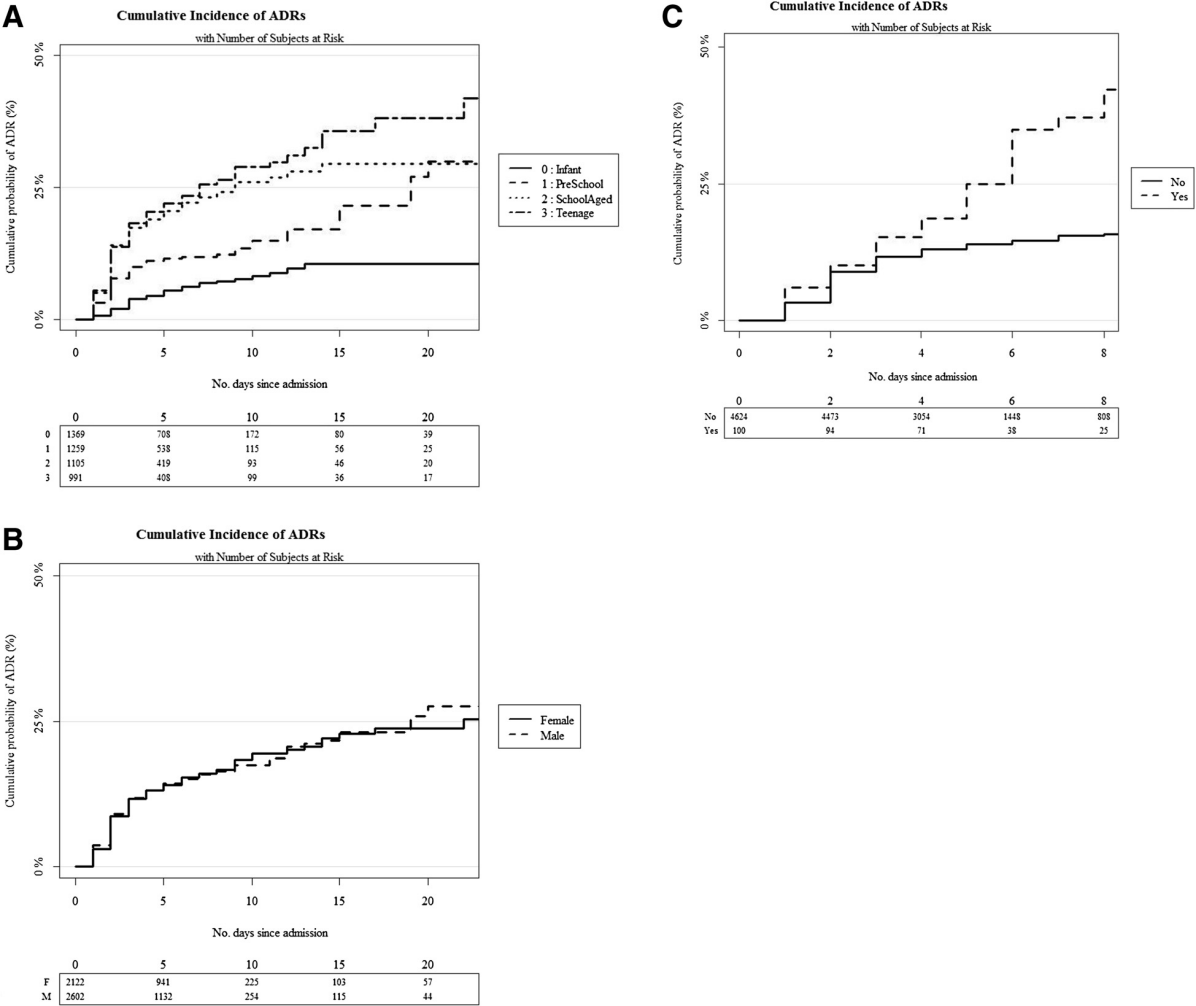
Cumulative incidence curves by categorical time invariant risk factor: (A) by age category, (B) gender and (C) oncology status.

The 10 most common reaction types together accounted for 76.6% of all ADRs [see Additional file 1: Table S1]. Pruritus, respiratory depression and urinary retention occurred almost exclusively in the post-anesthetic setting. In more than two-thirds of patients with nausea/vomiting, constipation or somnolence, drugs given during the anesthetic and/or used in post-operative pain management were implicated. Opioid analgesics and anesthetic agents were the most commonly implicated drug groups and accounted for 54% of all drugs associated with ADRs. Results of the univariate analysis are shown in Table 5 and Figure 2. Multivariate risk factor analysis of first admissions is shown in Table 6 and indicated that the risk of an ADR was associated with a GA, more than one medicine, being an oncology patient and age.

**Table 5:** Univariate analysis by categorical time invariant risk factor

**Covariate**		**Total patients**	**Number of patients with ADR**^**a**^	**Log rank statistic**
				**( *P *-value)**
Gender	Male	2,602	382	0.900
	Female	2,122	312	
Age	Infant (<1 years)	1,369	78	<0.001
	Pre-school (1 to 5 years)	1,259	155	
	School-aged (5 to 11 years)	1,105	231	
	Teenage (>11 years)	991	230	
Oncology	Yes	106	649	<0.001
	No	4,625	45	

^a^Only the first ADR was included in this analysis. *ADR* adverse drug reaction.

**Table 6:** Risk factors for ADRs assessed by multivariate analysis

**Covariate**		**HR (95% CI)**	***P*-value**
Age on admission (in years)		1.06 (1.04 to 1.07)	<0.001
Gender	Female	1	0.301
	Male	0.93 (0.80 to 1.08)	
Number of drugs		1.25 (1.22 to 1.28)	<0.001
Received a GA	No	1	<0.001
	Yes	6.38 (5.30 to 7.68)	
Oncology	No	1	<0.001
	Yes	1.89 (1.36 to 2.63)	

*ADRs* adverse drug reactions, *CI* confidence interval, *GA* general anesthesia, *HR* hazard ratio.

Most previous pediatric inpatient studies were carried out in general pediatric settings [8] in which only a small number of patients would have undergone GAs, thus underrepresenting drugs used in pediatric peri- and post-operative management. Rashed *et al.*[2] conducted a pediatric study on general medical wards and reported that anesthetics, which accounted for only 1% of all prescriptions, were among the drugs most commonly implicated in ADRs. In the two previous inpatient studies investigating pediatric surgical patients and providing medication details, opiate analgesics were among the two most commonly implicated drugs. However, GAs were not included, perhaps because they were not specifically investigated [19,20]. The differences in our study population, which included a large number of surgical patients (but not those admitted to the PICU immediately post-operatively), are also reflected in the spectrum and severity of common reaction types observed. Some reaction types such as urinary retention and respiratory depression/arrest occurred almost exclusively following GA. Eight of the 12 reactions classified as severe in our study occurred in post-operative patients and led to transfer to the HDU or PICU (Table 3). Notably, the risk of experiencing an ADR in patients undergoing a procedure under GA has not been assessed previously. In addition, our study confirmed risk factors that have been identified previously including increasing age, oncology treatment, and number of drugs [2,21]. It is not entirely clear why ADR risk increased with age, but is likely to be due to many factors including lack of detection and underreporting in younger or mentally disabled children (where symptoms dependent on patient communication, for example, nausea, pain, hallucinations, were underrepresented); acceptance of some common clinical manifestations such as vomiting and loose stools as being ‘normal’ in younger children; and reaction types such as pruritus being mistaken for ‘unsettledness’ in infants.

The observational approach depends on documentation by the clinical team regarding signs and symptoms and is, thus, a limitation of our study. A further limitation of the study was the exclusion of PICU patients, which is likely to have led to a lower overall ADR incidence. We deliberately excluded PICU patients because the causality assessment was more difficult and requires different methodologies for detection*.* Any ADRs occurring in patients admitted for less than 48 hours would also not have been included in this study. However, if such ADRs had required re-admission to hospital this would have been captured in our admissions study [21] and any ADRs requiring further management would have probably led to an extension of stay beyond 48 hours. Thus, although we would have missed ADRs in the time period of 0 to 48 hours, these were probably the less serious ADRs. Despite intense surveillance, it is possible that some ADRs were missed. The assessment of symptoms due to the underlying condition and differentiating these from those caused by drugs (for example, tachycardia in patients being treated for acute asthma) remains a challenge.

## Conclusions

Our data show that 17.7% of all children who spent more than 48 hours as an in-patient experienced at least one ADR. It is likely that our figures underestimated the true incidence of adverse events that should be attributed to drugs as we excluded ‘possible’ and ‘unlikely’ ADRs. A total of 58% of the ADRs observed in our study occurred in patients undergoing a procedure under GA which, at the same time, increased the risk of developing an ADR by more than six times. Our study did not assess children who stayed in hospital for less than 48 hours. ADRs may also be an important problem in children who are discharged home shortly after surgery. Given the current strategies to increase the proportion of children having day case surgery [22,23], this warrants further investigation.

Although less than 1% of reactions in our study were classified as severe, this does not take into account what impact an ADR might have on the child and/or the caregiver. For instance, a teenage patient is likely to feel very distressed about having to be catheterized because of urinary retention or receive an enema to treat constipation. The most common reaction in our study was vomiting, mainly observed in post-operative patients. Vomiting is a common and non-specific symptom in children and, thus, unlikely to be regarded as being particularly significant by clinicians. However, Diez reported that parents placed a very high value on the distress caused by postoperative vomiting [24]. In addition, parents of children included in this study reported that suspected ADRs cause them concern, irrespective of the ‘medical’ severity of the suspected reaction. On the other hand, parents valued the proactive explanations of ADRs given by oncologists and we suggest that a detailed discussion of this should form part of the preoperative assessment [25].

In conclusion, ADRs in hospitalized children are common and the incidence is much greater than in adults. Drugs used in perioperative management appear to be a major risk factor for experiencing an ADR, thus systematic monitoring of common and severe adverse effects of these drug groups would be an important step towards improving their safety.

## Abbreviations

ADRs: Adverse drug reactions; CI: Confidence interval; GA: General anesthesia; HDU: High dependency unit; HR: Hazard ratio; IQR: Interquartile range; PICU: Pediatric ICU.

## Competing interests

RLS and MPir are members of the Commission on Human Medicines. MPir chairs the Pharmacovigilance Expert Advisory Group, while RLS chairs the Paediatric Medicines Expert Advisory Group. RLS and MPir are senior NIHR investigators. The other authors declare they have no conflicts of interest. The views expressed in this article are solely those of the authors and not of any institutions that they represent. The funders had no role in study design, data collection, analysis, decision to publish, or preparation of the manuscript.

## Authors’ contributions

MP, AJN, MAT, MPir, RLS and PRW planned the study, were involved in study design and critically revised the manuscript. ST, JRB, LEB, HLM, KAB and JCD contributed to study design. ST developed the methods. ST and JRB developed the study protocol. ST, JRB, LEB, HLM, KAB and JCD collected data. JJK, EJC, LC and PRW designed the analysis plan. EJC conducted the analysis. RLS acts as guarantor and accepts responsibility for the integrity of the work as a whole. All authors read and approved the final manuscript.

## Pre-publication history

The pre-publication history for this paper can be accessed here:

http://www.biomedcentral.com/1741-7015/11/237/prepub

## Supplementary Material

Additional file 1: Table S1 Drug groups implicated in ADRs by frequency with associated reaction types. Click here for file
