# Supplementary material for: Incidence, characteristics and risk factors of adverse drug reactions in hospitalized children – a prospective observational cohort study of 6,601 admissions
Source: BMC Med. 2013 Nov 7;11:237. doi: 10.1186/1741-7015-11-237 (PMC4225679; doi:10.1186/1741-7015-11-237)
Supplement: Additional file 1: Table S1 — Drug groups implicated in ADRs by frequency with associated reaction types. [file 1741-7015-11-237-S1.pdf]

Additional file 1: Table S1. Drug groups implicated in ADRs by frequency with associated reaction types

Drug Group<br>(N of ADR cases)	Total N of drugs<br>(% of total)	Drugs (N)	ADR type[a] (N)
Opioid analgesics (688)	844 (27.9%)	Codeine (144)<br>Diamorphine (2)<br>Dihydrocodeine (4)<br>Fentanyl (267)<br>Morphine (426)<br>Tramadol (1)	Pruritus (198)<br>Nausea or Vomiting (186)<br>Constipation (143)<br>Respiratory arrest/ depression (3/37)<br>Somnolence without cardio-respiratory symptoms (37)<br>Urinary retention (28)<br>Myoclonus (13)<br>Hallucination (8)<br>Rash (4)<br>Bradycardia (3)<br>Dizziness (3)<br>Drug withdrawal syndrome (3)<br>Ileus (3)<br>Agitation (2)<br>Delayed recovery from anesthesia (2)<br>Flushing (2)<br>Visual disturbance (2)<br>Other[b] (11)
Drugs used in GA [excluding opiate analgesics other than remifentanyl] (322)	779 (25.8%)	Atracurium (4)<br>Atropine (1)<br>Desflurane (54)<br>Isoflurane (38)<br>Ketamine (6)<br>Nitrous oxide (131)<br>Propofol (200)<br>Remifentanil (83)<br>Rocuronium (4)<br>Sevoflurane (253)<br>Thiopental (4)<br>Vecuronium (1)	Nausea or vomiting (266)<br>Urinary retention (21)<br>Respiratory arrest or depression (2/6)<br>Delayed recovery from anesthesia (5)<br>Flushing (4)<br>Bradycardia (3)<br>Allergic reaction (3)<br>Hypotension (3)<br>Pruritus (2)<br>Other[b] (7)
Cytotoxic drugs and drugs used for cytotoxic induced side effects (179)	405 (13.4%)	Actinomycin D (5)<br>Allopurinol (4)<br>Amsacrine (2)<br>Asparaginase (6)<br>Busulfan (7)<br>Carboplatin (12)<br>Cisplatin (12)<br>Cladribine (1)<br>Clofarabine (5)<br>Cyclophosphamide (46)<br>Cytarabine (41)<br>Daunorubicin (13)<br>Doxorubicin (22)<br>Etoposide (56)<br>Fludarabine (6)<br>Gemcitabine (1)<br>Idarubicin (3)<br>Ifosfamide (21)<br>Irinotecan (1)	Nausea or vomiting (81)<br>Stomatitis (16)<br>Pancytopenia (13)<br>Diarrhea and vomiting (9)<br>Diarrhea without vomiting (9)<br>Hepatotoxicity (11; 8 increased transaminases only)[c]<br>Febrile neutropenia (6)<br>Rash (6)<br>Pain in jaw (3)<br>Constipation (3)<br>Pain other than jaw (2)<br>Headache (2)<br>Hyperglycemia (3)<br>Oral candidiasis (3)<br>Other[b] (14)

		Melphalan (11)<br>Mesna (15)<br>Methotrexate (31)<br>Mitoxantrone (4)<br>Rasburicase (4)<br>Temozolomide (2)<br>Thiotepa (3)<br>Tretinoin (1)<br>Vincristine (70)	
Antibiotics (162)	319 (10.6%)	Amikacin (3)<br>Amoxicillin (8)<br>Benzylpenicillin (4)<br>Cefalexin (17)<br>Cefotaxime (56)<br>Cefradine (1)<br>Ceftazidime (13)<br>Ceftriaxone (1)<br>Cefuroxime (18)<br>Ciprofloxacin (16)<br>Clarithromycin (7)<br>Clindamycin (2)<br>Co-amoxiclav (16)<br>Co-trimoxazole (3)<br>Flucloxacillin (15)<br>Gentamicin (29)<br>Meropenem (4)<br>Metronidazole (29)<br>Penicillin V (5)<br>Piperacillin and Tazobactam (28)<br>Rifampicin (10)<br>Teicoplanin (19)<br>Tobramycin (3)<br>Trimethoprim (3)<br>Vancomycin (7)	Diarrhea (66)<br>Candidiasis (38)<br>Rash (16)<br>Nausea or vomiting (8)<br>Clostridium difficile colitis (7)<br>Colonization with Candida (4)<br>Transaminases increased (4)<br>Anaphylactic reaction(2)<br>Angioedema (2)<br>Flushing (2)<br>Hepatotoxicity (3)[c]<br>Pruritus (2)<br>Other[b] (8)
Drugs used in epidurals regional anesthetics and IV drugs used in post-operative pain management other than opioids (188)	195 (6.4%)	Clonidine & Levobupivicaine (25)<br>Clonidine (7)<br>Fentanyl & Levobupivicaine (116)<br>Ketamine (36)<br>Levobupivacaine (11)	Pruritus (52)<br>Nausea and/or Vomiting (35)<br>Constipation (24)<br>Urinary retention (15)<br>Somnolence without cardio-respiratory symptoms (11)<br>Respiratory depression/arrest (8/1)<br>Hypotension (7)<br>Paresthesia (6)<br>Bradycardia (4)<br>Myoclonus (3)<br>Hypoesthesia (2)<br>Visual disturbance (2)<br>Hallucination (2)<br>Hypertension (2)<br>Urinary incontinence (2)<br>Other[b] (12)

Corticosteroids (51)	62 (2.1%)	Beclomethasone (1)<br>Dexamethasone (24)<br>Fludrocortisone (1)<br>Hydrocortisone (8)<br>Methylprednisolone (14)<br>Prednisolone (14)	Hyperglycemia (13)<br>Hypertension (8)<br>Candidiasis (9)<br>Fluid retention (2)<br>Gastritis (2)<br>Other[b] (17)
Bronchodilators (31)	58 (1.9%)	Aminophylline (21)<br>Ipratropium (2)<br>Salbutamol (35)	Hypokalemia (15)<br>Nausea and/or vomiting (7)<br>Tremor (4)<br>Tachycardia (2)<br>Other[b] (3)
Antiemetics (50)	55 (1.8%)	Cyclizine (1)<br>Levomepromazine (3)<br>Ondansetron (51)	Constipation (45)<br>Disorientation (2)<br>Other[b] (4)
Antiepileptic drugs (45)	49 (1.6%)	Carbamazepine (3)<br>Diazepam (3)<br>Gabapentin (2)<br>Lorazepam (1)<br>Midazolam (35)<br>Pregabalin (4)<br>Valproate (1)	Nausea and/or vomiting (24)<br>Somnolence without cardio-respiratory symptoms (6)<br>Abnormal behavior (2)<br>Constipation (2)<br>Delayed recovery from anesthesia (2)<br>Respiratory depression (2)<br>Other[b] (7)
Diuretics (28)	41 (1.4%)	Chlorothiazide (1)<br>Furosemide (30)<br>Metolazone (2)<br>Spironolactone (8)	Hypernatremia (9)<br>Hypokalemia (8)<br>Hypotension (3)<br>Hypomagnesaemia (4)<br>Other[b] (5)
Drugs affecting the immune response (suppression and modulation) + cytokine modulators (31)	34 (1.1%)	Adesleukin (5)<br>Alemtuzumab (11)<br>Azathioprine (1)<br>Ciclosporin (7)<br>Mycophenolate (1)<br>Rabbit anti-human thymocyte immunoglobulin (3)<br>Rituximab (2)<br>Tacrolimus (3)<br>Tocilizumab (1)	Pyrexia (4)<br>Candidiasis (4)<br>Infusion associated reaction (3)<br>Stomatitis (3)<br>Edema (2)<br>Pruritus (2)<br>Vomiting (2)<br>Other[b] (11)
Drugs affecting the cardiovascular system (23)	27 (0.9%)	Amlodipine (4)<br>Bisoprolol (1)<br>Captopril (10)<br>Carvedilol (1)<br>Dinoprostone (1)<br>Enalapril (1)<br>Hydralazine (1)<br>Isoprenaline (1)<br>Lisinopril (4)<br>Milrinone (3)	Hypotension (11)<br>Hyperglycemia and Glycosuria (3)<br>Other[b] (9)
NSAIDS (+ aspirin) (24)	24 (0.8%)	Aspirin (2)<br>Diclofenac (15)<br>Ibuprofen (5)<br>Naproxen (2)	Nausea and/or vomiting (11)<br>Hematemesis (3)<br>Other gastrointestinal bleed (2)<br>Constipation (2)<br>Other[b] (5)

Laxatives (20)	22 (0.7%)	Docusate (3,<br>Lactulose (12)<br>Macrogol (6)<br>Sennoside (1)	Diarrhea (17)<br>Abdominal pain (2)<br>Vomiting (1)
Antifungals and Antivirals (20)	21 (0.7%)	Aciclovir (5)<br>Amphotericin (7)<br>Fluconazole (4)<br>Itraconazole (1)<br>Miconazole (1)<br>Ribivarin (1)<br>Voriconazole (2)	Diarrhea (8)<br>Hepatotoxicity[c] (3)<br>Hypokalemia (3)<br>Other[b] (5)
Drugs used in diabetes and hypoglycemia (13)	16 (0.5%)	Diazoxide (3)<br>Glucagon (1)<br>Insulin (4)<br>Insulin aspart (4)<br>Insulin detemir (4)	Hypoglycemia (7)<br>Fluid overload (2)<br>Hypokalemia (2)<br>Other[b]
Other (69)	73 (2.4%)	-	-

[a]If the same patient experienced two types of reactions to the same medication(s) at the same time this would have been reported as one ADR case but will be listed here as two reaction types, for example, a patient with respiratory depression and bradycardia = one ADR case, but is listed as two reactions;
[b]Reactions that occurred only once are listed as 'other';
[c]Transaminases were raised in all cases. Additionally, if other parameters of liver function such as bilirubin and INR were also raised, we classified this as hepatotoxicity. ADR adverse drug reaction; GA general anesthesia; N number; INR international normalized ratio.
